# Association of Maternal History of Preterm Birth With Congenital Anomalies in Offspring

**DOI:** 10.1001/jamanetworkopen.2022.23614

**Published:** 2022-07-25

**Authors:** Ran Wang, Chao Chen, Jiaxi Huang, Bing Jia, Qiqi Shi

**Affiliations:** 1 Department of Neonatology, Children’s Hospital of Fudan University, National Children’s Medical Center, Shanghai, China; 2 NHC Key Laboratory of Neonatal Diseases, Fudan University, Shanghai, China; 3 Department of Pediatric Cardiothoracic Surgery, Children’s Hospital of Fudan University, National Children’s Medical Center, Shanghai, China; 4 Kunshan Maternity and Children's Health Care Hospital, Kunshan, China

## Abstract

This study examines the prevalence of congenital anomalies in infants born to mothers with preterm birth occurring in a previous pregnancy.

## Introduction

Congenital anomalies (CAs) are the leading cause of infant mortality, accounting for more than 20% of infant deaths in the US in 2017.^1^ Several parental risk factors of CAs, including diabetes before pregnancy and maternal smoking, have been identified.^2,3^ However, data on the associations of maternal history of previous pregnancy outcomes with the risk of CAs are sparse. To further elucidate potential risk factors, we evaluated maternal history of preterm birth (PTB) and offspring CAs.

## Methods

This retrospective population-based cohort study used birth data from the US National Vital Statistics System from January 1, 2016, to December 31, 2019, including all women with a live singleton birth. Data analysis was conducted from February 1 to February 15, 2022. Because deidentified data are publicly available, ethics approval was not required by the institutional review board of Children’s Hospital of Fudan University. This study is reported following the Strengthening the Reporting of Observational Studies in Epidemiology (STROBE) reporting guideline.

Information on maternal history of PTB and 12 subtypes of CAs was retrieved from birth certificates. Because of low incidence of the outcome, proportions are expressed in parts per thousand (‰). The associations of maternal history of PTB with neonatal CAs were estimated as risk differences, crude odds ratios (ORs), and adjusted ORs (aOR) with 95% CIs. Stratified analyses according to baseline characteristics were performed. Adjustments were made for potential confounders, including maternal age, race and ethnicity, educational levels, marital status, parity, smoking before and during pregnancy, prepregnancy body mass index categories, timing of initiation of prenatal care, prepregnancy hypertension, prepregnancy diabetes, gestational hypertension, gestational diabetes, and infant sex. A description of methods and potential confounders is available in the eMethods in the Supplement. All *P* values were 2-sided, and *P* < .05 was considered statistically significant. Statistical analyses were performed using Stata, version 15.0 (StataCorp LLC).

## Results

A total of 14 774 946 mother-neonate pairs with live singleton birth were included in the final analysis; the mean (SD) age of the mothers was 28.86 (5.81) years. The prevalence of CAs was 3.19‰ (47 205 of 14 774 946). Neonates born to mothers with a history of PTB had a higher prevalence in parts per thousand of CAs than did neonates born to mothers without a PTB history (5.25‰ [2554 of 486 894] vs 3.13‰ [44 651 of 14 288 052]; risk difference, 2.12; 95% CI, 1.91-2.33; crude OR, 1.68; 95% CI, 1.62-1.75). After full adjustment, the OR of CAs was 1.47 (95% CI, 1.42-1.54) for maternal history of PTB. For specific subtypes of CAs, maternal history of PTB was associated with an increased risk of nearly all subtypes except anencephaly (aOR, 1.25; 95% CI, 0.87-1.79). For example, the aOR of cyanotic congenital heart disease was 1.76 (95% CI, 1.60-1.93) for maternal history of PTB (Table 1).

**Table 1.** Association of Maternal History of PTB With Congenital Anomalies in Offspring

Congenital anomalies	No maternal history of PTB (n = 14 288 052)	Maternal history of PTB (n = 486 894)
Congenital anomalies^a^		
Cases, No. (‰)	44 651 (3.13)	2 554 (5.25)
RD (95% CI), ‰	1 [Reference]	2.12 (1.91 to 2.33)
Crude OR (95% CI)	1 [Reference]	1.68 (1.62 to 1.75)
aOR (95% CI)^b^	1 [Reference]	1.47 (1.42 to 1.54)
Cyanotic congenital heart disease		
Cases, No. (‰)	6 959 (0.49)	527 (1.08)
RD (95% CI), ‰	1 [Reference]	0.60 (0.50 to 0.69)
Crude OR (95% CI)	1 [Reference]	2.22 (2.04 to 2.43)
aOR (95% CI)^b^	1 [Reference]	1.76 (1.60 to 1.93)
Congenital diaphragmatic hernia		
Cases, No. (‰)	1 414 (0.10)	87 (0.18)
RD (95% CI), ‰	1 [Reference]	0.08 (0.04 to 0.12)
Crude OR (95% CI)	1 [Reference]	1.81 (1.45 to 2.24)
aOR (95% CI)^b^	1 [Reference]	1.76 (1.41 to 2.21)
Omphalocele		
Cases, No. (‰)	1 185 (0.08)	63 (0.13)
RD (95% CI), ‰	1 [Reference]	0.05 (0.01 to 0.08)
Crude OR (95% CI)	1 [Reference]	1.56 (1.21 to 2.01)
aOR (95% CI)^b^	1 [Reference]	1.60 (1.23 to 2.08)
Gastroschisis		
Cases, No. (‰)	3 130 (0.22)	121 (0.25)
RD (95% CI), ‰	1 [Reference]	0.03 (−0.02 to 0.07)
Crude OR (95% CI)	1 [Reference]	1.13 (0.95 to 1.36)
aOR (95% CI)^b^	1 [Reference]	1.76 (1.46 to 2.13)
Limb reduction defect		
Cases, No. (‰)	1 650 (0.12)	89 (0.18)
RD (95% CI), ‰	1 [Reference]	0.07 (0.03 to 0.01)
Crude OR (95% CI)	1 [Reference]	1.58 (1.28 to 1.96)
aOR (95% CI)^b^	1 [Reference]	1.43 (1.14 to 1.78)
Cleft lip with or without cleft palate		
Cases, No. (‰)	6 938 (0.49)	349 (0.72)
RD (95% CI), ‰	1 [Reference]	0.23 (0.16 to 0.31)
Crude OR (95% CI)	1.00 [Reference]	1.48 (1.33 to 1.64)
aOR (95% CI)^b^	1.00 [Reference]	1.32 (1.18 to 1.47)
Cleft palate alone		
Cases, No. (‰)	3 161 (0.22)	177 (0.36)
RD (95% CI), ‰	1 [Reference]	0.14 (0.09 to 0.20)
Crude OR (95% CI)	1 [Reference]	1.64 (1.41 to 1.91)
aOR (95% CI)^b^	1 [Reference]	1.41 (1.20 to 1.65)
Hypospadias		
Cases, No. (‰)	8 136 (1.11)	394 (1.59)
RD (95% CI), ‰	1 [Reference]	0.48 (0.32 to 0.63)
Crude OR (95% CI)	1 [Reference]	1.43 (1.29 to 1.58)
aOR (95% CI)^b^	1 [Reference]	1.45 (1.30 to 1.61)
Anencephaly		
Cases, No. (‰)	684 (0.05)	33 (0.07)
RD (95% CI), ‰	1 [Reference]	0.02 (−0.004 to 0.04)
Crude OR (95% CI)	1 [Reference]	1.42 (1.00 to 2.01)
aOR (95% CI)^b^	1 [Reference]	1.25 (0.87-1.79)
Meningomyelocele/spina bifida		
Cases, No. (‰)	1 848 (0.13)	109 (0.22)
RD (95% CI), ‰	1 [Reference]	0.09 (0.05 to 0.14)
Crude OR (95% CI)	1 [Reference]	1.73 (1.43 to 2.10)
aOR (95% CI)^b^	1 [Reference]	1.55 (1.27 to 1.90)
Down syndrome		
Cases, No. (‰)	7 319 (0.51)	473 (0.97)
RD (95% CI), ‰	1 [Reference]	0.46 (0.37 to 0.55)
Crude OR (95% CI)	1 [Reference]	1.90 (1.73 to 2.08)
aOR (95% CI)^b^	1 [Reference]	1.30 (1.18 to 1.43)
Suspected chromosomal disorder		
Cases, No. (‰)	5 137 (0.36)	356 (0.73)
RD (95% CI), ‰	1 [Reference]	0.37 (0.30 to 0.45)
Crude OR (95% CI)	1 [Reference]	2.03 (1.83 to 2.57)
aOR (95% CI)^b^	1 [Reference]	1.66 (1.48 to 1.95)

Abbreviations: aOR, adjusted odds ratio; PTB, preterm birth; RD, risk difference.

^a^ Congenital anomalies defined as any specific subtype of congenital anomalies in the data set.

^b^ Adjusted for maternal age group, race and ethnicity, educational levels, marital status, parity, smoking before pregnancy and during pregnancy, prepregnancy body mass index categories, timing of initiation of prenatal care, prepregnancy hypertension, prepregnancy diabetes, gestational hypertension, gestational diabetes, and infant sex (except for hypospadias).

Subgroup analyses by maternal age, race and ethnicity, educational level, parity, smoking before and during pregnancy, time of initiation of prenatal care, prepregnancy body mass index, prepregnancy hypertension, prepregnancy diabetes, gestational hypertension, gestational diabetes, and neonate sex were conducted. The neonates who were born to mothers with a history of PTB had a higher risk of CAs in all subgroups after full adjustment (Table 2).

**Table 2.** Subgroup Analyses of Associations Between Maternal History of PTB and Risk of Congenital Anomalies in Offspring

Variable	No history of PTB	Maternal history of PTB^a^			
	No. cases/No. total	No. cases/No. total	RD vs reference (95% CI), ‰	Crude OR (95% CI)	aOR (95% CI)^b^
Maternal age group, y					
<30	23 122/7 741 276	1 042/220 660	1.74 (1.45-2.02)	1.58 (1.49-1.69)	1.59 (1.49-1.69)
30-34	11 103/4 054 751	700/150 651	1.91 (1.56-2.26)	1.70 (1.57-1.84)	1.48 (1.37-1.61)
35-39	7 297/2 041 220	541/92 699	2.26 (1.76-2.76)	1.64 (1.50-1.79)	1.37 (1.25-1.50)
≥40	3 129/450 805	271/22 884	4.90 (3.48-6.32)	1.71 (1.51-1.94)	1.38 (1.22-1.57)
Race and ethnicity^c^					
Hispanic	9 016/3 402 006	514/106 969	2.15 (1.74-2.57)	1.82 (1.66-1.99)	1.49 (1.36-1.64)
Non-Hispanic Black	4 916/2 010 484	404/101 911	1.52 (1.13-1.91)	1.62 (1.47-1.80)	1.46 (1.31-1.62)
Non-Hispanic White	26 952/7 368 270	1 426/236 111	2.38 (2.07-2.70)	1.66 (1.57-1.75)	1.47 (1.40-1.56)
Other^d^	3 425/1 383 200	190/38 259	2.49 (1.78-3.20)	2.01 (1.74-2.33)	1.49 (1.28-1.73)
Missing data	342/124 092	20/3 544	2.73 (0.32-5.15)	2.00 (1.27-3.14)	1.37 (0.86-2.19)
Educational level					
<High school diploma	6 448/1 833 168	472/83 178	2.16 (1.64-2.67)	1.62 (1.47-1.78)	1.46 (1.32-1.60)
High school diploma	11 800/3 613 829	710/140 464	1.79 (1.41-2.16)	1.55 (1.44-1.67)	1.45 (1.34-1.57)
>High school diploma	25 910/8 657 434	1 338/258 050	2.19 (1.91-2.47)	1.74 (1.64-1.83)	1.50 (1.42-1.59)
Missing data	493/183 621	34/5 202	3.85 (1.65-6.05)	2.44 (1.72-3.46)	1.68 (1.17-2.42)
Parity					
1	12 739/4 543 797	771/166 601	1.82 (1.49-2.15)	1.65 (1.54-1.78)	1.47 (1.37-1.59)
2	7 410/2 355 674	743/146 467	1.93 (1.56-2.30)	1.62 (1.50-1.74)	1.49 (1.38-1.61)
3	3 407/989 607	497/87 745	2.22 (1.71-2.73)	1.65 (1.50-1.81)	1.56 (1.41-1.71)
≥4	2 917/680 685	539/84 704	2.08 (1.52-2.64)	1.45 (1.36-1.63)	1.49 (1.36-1.64)
Missing data	85/37 446	4/1 318	0.77 (−2.24-3.77)	1.34 (0.49-3.65)	1.21 (0.44-3.33)
Smoking before pregnancy					
No	38 981/13 026 560	2 058/410 724	2.02 (1.80-2.24)	1.68 (1.60-1.75)	1.48 (1.41-1.55)
Yes	5 425/1 195 369	472/72 683	1.96 (1.36-2.55)	1.43 (1.30-1.58)	1.46 (1.32-1.61)
Missing data	245/66 123	24/3 487	3.18 (0.39-5.96)	1.86 (1.22-2.83)	1.91 (1.23-2,97)
Smoking during pregnancy					
No	40 041/13 292 525	2 113/419 453	2.03 (1.81-2.24)	1.68 (1.60-1.75)	1.49 (1.42-1.56)
Yes	4 255/913 305	404/61 968	1.86 (1.21-2.51)	1.40 (1.27-1.55)	1.45 (1.30-1.61)
Missing data	355/82 222	37/5 473	2.44 (0.23-4.66)	1.57 (1.12-2.20)	1.37 (0.96-1.97)
Time of initiation of prenatal care					
First to third mo	30 883/10 785 358	1 644/346 936	1.88 (1.64-2.11)	1.66 (1.58-1.74)	1.44 (1.37-1.52)
Fourth to sixth mo	8 604/2 274 797	573/90 735	2.53 (2.01-3.05)	1.67 (1.54-1.82)	1.55 (1.42-1.70)
Seventh to ninth mo	2 781/641 303	162/24 156	2.37 (1.33-3.41)	1.55 (1.32-1.82)	1.48 (1.26-1.74)
No prenatal care	849/234 780	70/11 969	2.23 (0.84-3.62)	1.62 (1.27-2.07)	1.44 (1.12-1.85)
Missing data	1 534/351 814	105/12 993	3.66 (2.11-5.20)	1.85 (1.51-2.25)	1.62 (1.32-1.99)
Prepregnancy BMI					
18.5-24.9	18 030/6 010 519	937/174 759	2.36 (2.02-2.71)	1.79 (1.68-1.92)	1.56 (1.45-1.66)
<18.5	1 430/461 232	91/15 979	2.59 (1.42-3.77)	1.84 (1.49-2.28)	1.67 (1.33-2.09)
25.0-29.9	11 375/3 689 966	601/125 146	1.72 (1.33-2.10)	1.56 (1.44-1.69)	1.37 (1.26-1.49)
≥30	12 628/3 783 718	845/158 615	1.99 (1.63-2.35)	1.60 (1.49-1.72)	1.44 (1.34-1.54)
Missing data	1 188/342 617	80/12 395	2.99 (1.56-4.41)	1.87 (1.49-2.34)	1.51 (1.19-1.91)
Prepregnancy hypertension					
No	43 322/14 027 747	2 360/462 419	2.02 (1.81-2.22)	1.66 (1.59-1.73)	1.47 (1.41-1.54)
Yes	1 329/260 305	194/24 475	2.82 (1.68-3.97)	1.56 (1.34-1.81)	1.51 (1.29-1.77)
Gestational hypertension					
No	40 756/13 361 262	2 255/438 335	2.09 (1.88-2.31)	1.69 (1.62-1.76)	1.49 (1.43-1.56)
Yes	3 895/926 790	299/48 559	1.96 (1.25-2.66)	1.47 (1.30-1.65)	1.33 (1.17-1.50)
Prepregnancy diabetes					
No	43 734/14 165 096	2 394/474 947	1.95 (1.75-2.16)	1.64 (1.57-1.70)	1.46 (1.40-1.53)
Yes	917/122 956	160/11 947	5.93 (3.82-8.05)	1.81 (1.53-2.14)	1.69 (1.41-2.02)
Gestational diabetes					
No	41 057/13 391 043	2 275/440 708	2.10 (1.88-2.31)	1.69 (1.62-1.76)	1.49 (1.43-1.56)
Yes	3 594/897 009	279/46 186	2.03 (1.32-2.75)	1.51 (1.34-1.71)	1.33 (1.17-1.51)
Neonate sex					
Male	27 692/7 310 500	1 539/248 117	2.41 (2.10-2.73)	1.64 (1.56-1.73)	1.48 (1.40-1.56)
Female	16 959/6 977 552	1 015/238 777	1.82 (1.55-2.08)	1.75 (1.64-1.87)	1.47 (1.38-1.57)

Abbreviations: aOR, adjusted odds ratio; BMI, body mass index (calculated as weight in kilograms divided by height in meters squared); OR, odds ratio; PTB, preterm birth.

^a^ Neonates born to mothers with no history of PTB were the reference group.

^b^ Adjusted for maternal age group, race and ethnicity, educational levels, marital status, parity, smoking before pregnancy and during pregnancy, prepregnancy BMI categories, timing of initiation of prenatal care, prepregnancy hypertension, prepregnancy diabetes, gestational hypertension, gestational diabetes, and infant sex were adjusted, except when the variable was stratified.

^c^ Self-reported.

^d^ Included individuals who were non-Hispanic Asian, non-Hispanic Native American or Alaskan, non-Hispanic Native Hawaiian or other Pacific Islanders, non-Hispanic people of more than 1 race, of unknown racial or ethnic origin, or not stated.

## Discussion

The findings of this study suggest that maternal history of PTB increased the risk of birth CAs in offspring. The mechanisms underlying the association between maternal history of PTB and birth CAs are yet to be elucidated. Previous PTB may be related to defects of the placenta and metabolic disorders of the mothers,^4,5^ which may involve the development of CAs. Limitations of this study include potential unmeasured confounding factors. Neonates born to mothers with a history of PTB may have an increased risk of CAs. These findings may help to identify neonates at high risk of CAs.

## Notes

Supplement. **eMethods.** Detailed Methods

## References

[1] Ely DM, Driscoll AK. Infant mortality in the United States, 2017: data From the Period Linked Birth/Infant Death File. Natl Vital Stat Rep. 2019;68(10):1-20. 32501205

[2] Wu Y, Liu B, Sun Y, . Association of maternal prepregnancy diabetes and gestational diabetes mellitus with congenital anomalies of the newborn. Diabetes Care. 2020;43(12):2983-2990. doi:10.2337/dc20-0261 33087319 PMC7770264

[3] Yang L, Wang H, Yang L, . Maternal cigarette smoking before or during pregnancy increases the risk of birth congenital anomalies: a population-based retrospective cohort study of 12 million mother-infant pairs. BMC Med. 2022;20(1):4. doi:10.1186/s12916-021-02196-x 35012532 PMC8750764

[4] Auger N, Potter BJ, He S, Healy-Profitós J, Schnitzer ME, Paradis G. Maternal cardiovascular disease 3 decades after preterm birth: longitudinal cohort study of pregnancy vascular disorders. Hypertension. 2020;75(3):788-795. doi:10.1161/HYPERTENSIONAHA.119.14221 32008431

[5] Romero R, Dey SK, Fisher SJ. Preterm labor: one syndrome, many causes. Science. 2014;345(6198):760-765. doi:10.1126/science.1251816 25124429 PMC4191866

