# Supplementary material for: Association of Maternal History of Preterm Birth With Congenital Anomalies in Offspring
Source: JAMA Netw Open. 2022 Jul 25;5(7):e2223614. doi: 10.1001/jamanetworkopen.2022.23614 (PMC9315420; doi:10.1001/jamanetworkopen.2022.23614)
Supplement: Supplement. — eMethods. Detailed Methods [file jamanetwopen-e2223614-s001.pdf]

Supplementary Online Content

Wang R, Chen C, Huang J, Jia B, Shi Q. Association of maternal history of preterm birth with congenital anomalies in offspring. *JAMA Netw Open*. 2022;5(7):e2223614. doi:10.1001/jamanetworkopen.2022.23614

eMethods. Detailed Methods

This supplementary material has been provided by the authors to give readers additional information about their work.

© 2022 Wang R et al. *JAMA Network Open.*

eMethods.

Study Population

This is nationwide cohort study using birth data in the US National Vital Statistics System (NVSS). This study included all live singleton birth from 2016 to 2019 in the U.S. Records with no information about previous history of preterm birth (PTB) or congenital anomalies (CAs) of offspring were excluded. The children's hospital of Fudan University institutional review board waived the need for review because these records are publicly available and did not involve human subjects. This study is reported following the Strengthening the Reporting of Observational Studies in Epidemiology (STROBE) reporting guideline.

Exposure and Outcome

The exposure was maternal previous history of PTB and collected from birth certificate. The main outcome was CAs of neonates. Twelve subtypes of CAs were collected directly from the medical records, including cyanotic congenital heart disease, congenital diaphragmatic hernia, cleft lip with or without cleft palate, gastroschisis, cleft palate alone, meningomyelocele/spina bifida, limb reduction defect, anencephaly, omphalocele, hypospadias (restricted to male neonates), Down syndrome, and suspected chromosomal disorder. In this study, CAs were defined as having any subtype of the above anomalies.

Covariables

Potential confounders considered for adjustment in the analysis included maternal age, educational level, marital status, self-reported race and ethnicity, prepregnancy body mass index (BMI), timing of initiation of perinatal care, smoking before and during pregnancy, prepregnancy hypertension, prepregnancy diabetes, gestational hypertension, gestational diabetes, and neonatal sex.

© 2022 Wang R et al. *JAMA Network Open.*

Maternal age was defined as age at the time of delivery and classified as younger than 30, 30 to 34, 35 to 39, or 40 years or older. Maternal educational levels were classified as lower than high school diploma, high school diploma, or higher than high school diploma. Maternal race and ethnicity were self-reported in the database and included Hispanic, non-Hispanic black, non-Hispanic white, and others (including non-Hispanic Asians, non-Hispanic Native American or Alaskans, non-Hispanic Native Hawaiians or other Pacific Islanders, non-Hispanic people of more than one race, people of unknown racial or ethnic origin, or not stated). Marital status was categorized as married or unmarried. Parity, defined as the total number of live births before this delivery, was classified as 0, 1, 2, 3, or 4 or more. Time of initiation of prenatal care was categorized by the trimester of the first prenatal visit as no prenatal care, first to third month, fourth to sixth month, and seven to ninth month. Smoking before pregnancy and during pregnancy was classified as "yes" and "no". Maternal prepregnancy BMI was classified as underweight (<18.5), normal weight (18.5-24.9), overweight (25.0-29.9), obesity (≥30). Prepregnancy diabetes and hypertension, gestational diabetes and hypertension were each categorized as yes, or no. Neonatal sex was categorized as male and female. A missing category for covariates was added when necessary.

Statistical analysis

Statistical analyses were performed using Stata, version 15.0 (StataCorp LLC). All P values were 2-sided, and $P < 0.05$ was considered statistically significant. Frequencies and percentage were reported for categorical variables. The associations of maternal history of PTB with neonatal CAs were estimated as absolute risk differences, crude, and adjusted odds ratio (aOR) with 95% confidence intervals (CI). Adjustments were made for potential confounders including maternal age group, race and ethnicity, educational levels, marital status, parity, smoking before pregnancy

and during pregnancy, prepregnancy BMI categories, timing of initiation of prenatal care, prepregnancy hypertension, prepregnancy diabetes, gestational hypertension, gestational diabetes, and infant sex.

In the stratified analysis, we divided women into different subgroups according to baseline characteristics (including maternal age, race and ethnicity, educational levels, pre-pregnancy BMI, timing of initiation of prenatal care, smoking before and during pregnancy, prepregnancy hypertension, prepregnancy diabetes, gestational hypertension, gestational diabetes, and neonatal sex). Among these subgroups, we analyzed the association between maternal history of PTB and CAs after adjusting for other potential risk factors to explore potential disparities in the association between PTB and CAs.

© 2022 Wang R et al. *JAMA Network Open.*
